# Stochastic phase segregation on surfaces

**DOI:** 10.1098/rsos.170472

**Published:** 2017-08-16

**Authors:** Prerna Gera, David Salac

**Affiliations:** Department of Mechanical and Aerospace Engineering, University at Buffalo, Buffalo, NY 14260-4400, USA

**Keywords:** Cahn–Hilliard, stochastic, curved surfaces, phase field, probability distribution

## Abstract

Phase separation and coarsening is a phenomenon commonly seen in binary physical and chemical systems that occur in nature. Often, thermal fluctuations, modelled as stochastic noise, are present in the system and the phase segregation process occurs on a surface. In this work, the segregation process is modelled via the Cahn–Hilliard–Cook model, which is a fourth-order parabolic stochastic system. Coarsening is analysed on two sample surfaces: a unit sphere and a dumbbell. On both surfaces, a statistical analysis of the growth rate is performed, and the influence of noise level and mobility is also investigated. For the spherical interface, it is also shown that a lognormal distribution fits the growth rate well.

## 1. Introduction

Domains on curved surfaces are found in numerous industrial and biomedical applications such as chemical reactors [1], enhanced oil recovery [2] and pulmonary functions [3]. These domains have the potential to change the dynamics of these systems significantly. For example, surfactants on bubbles or droplets can reduce the velocity of the rising bubble [4] or they can prevent the coalescence of multiple bubbles [1]. The effect of surface molecules can also be seen in the area of biology such as the cell membrane [5]. The cell membrane is composed of multiple components including saturated lipids, unsaturated lipids and cholesterol. The saturated lipid molecules combine with the cholesterol to form lipid domains, with these lipid domains being more ordered and stable than the surrounding membrane [6]. Owing to the nature of the domains, experimental visualization is difficult with artefacts and errors influencing the accuracy of the experimental results. Using numerical tools and mathematical modelling to investigate the dynamics of surface domains can provide important information not obtainable experimentally. In addition to biological membranes, other interesting phase dynamics on a curved surface includes crystal growth [7], phase separation within thin films [8] and phase separation patterns in diblock polymers [9]. With this motivation, the goal of this work is to study the phase segregation dynamics on a smooth curved surface.

The Cahn–Hilliard (CH) equation is a popular model to capture and investigate phase segregation dynamics in a multicomponent system. It describes the temporal evolution of an order parameter that defines the phase or domain, with the driving force given by energy minimization under the assumption of quantity conservation. Pioneered by Cahn & Hilliard [10], the equation has been used to model many physical systems including binary alloys [10], polymers and ceramics [11], droplet break-up [12], liquid–liquid jets pinching off [13], multicomponent lipid vesicles [14], and for the tracking of tumour growth [15].

In 1970, Cook proposed to make the system more realistic by including internal thermal fluctuations, which are represented by a conserved noise source term. This extension is more commonly known as the Cahn–Hilliard–Cook (CHC) model [16]. The first numerical work to study the CHC equation was done by Langer [17]. This work was compared against theoretical results and it was concluded that the thermal fluctuations play an important role in the early stage of phase dynamics [17]. To understand the early stages better, Grant *et al*. [18] developed a perturbation theory for a long range force limit and used numerical methods to investigate the model. Rodgers *et al.* [19] studied convergence of the solution, the growth characteristics of the domain formation and the effect of noise. The impact of noise in the CHC has also been analysed by several other works; see [20–22], for examples. Using this model, the influence of non-equilibrium lipid transport on a membrane [23], dendritic branching [24], nucleation in microstructures [25] and the dynamics of solvent-based organic cells [26] have also been investigated.

The CHC model can be solved computationally using a variety of numerical methods including finite differences [27–29], finite elements [30–32] and spectral methods [33,34]. Most investigations using the CHC model are two-dimensional, although there are some which investigate three-dimensional systems [22]. In this work, a method to model the CHC system on an arbitrary two-dimensional surface in three-dimensional space is presented. Prior works in modelling the phase segregation on curved surfaces have been performed at the nanometer level, including those based on molecular dynamics (MD) [35–37], where atomistic-level forces can be incorporated. A limit of these MD simulations is the length and timescales which can be investigated. To allow for longer timescales and larger domain sizes to be investigated, coarse-grained methods such as dissipative particle dynamics have been used [38]. An alternate technique is to examine phase segregation on surfaces using a continuum-based method, which is the approach taken here. Samples of this type of work in the absence of noise have been presented in the past [14,39–41].

The work here is based on a splitting method previously used to model the CH equation [42,43]. The coarsening rates for the CH and CHC systems are compared using both constant and variable mobility, in addition to varying noise levels. While in an actual system thermal fluctuations may also influence the shape of the underlying interface, this is not considered here.

In the following section of the paper, the governing equations of the system is described. The numerical tools and techniques that are used and the overall algorithm is then explained. Phase segregation on a sphere is examined and systematically investigated. A statistical analysis on the growth rate of the domains that appear on the sphere is carried out. Further, the effect of the underlying geometry is also shown as the statistical analysis of the growth rate on a dumbbell is presented.

## 2. Mathematical formulation

In this work, the dynamics of phase separation and coarsening of a two-component system on a surface is described by the CH equation. This equation captures how a system will change over time to reduce the overall free energy of a multicomponent system. Further, the segregation process is restricted to a co-dimension one interface and thus will involve surface derivatives. Let *Γ*(***x***,*t*) define the interface at any point in time. The concentration field *f*(***x***,*t*) is defined on *Γ*(***x***,*t*) such that 0≤*f*(***x***,*t*)≤1 is the concentration of one surface phase, while the concentration of the remaining surface phase is 1−*f*(***x***,*t*). Using this, the CH equation is derived from the mass continuity equation:

2.1 $$\frac{\partial f}{\partial t}+\nabla_{s}\cdot J_{s}=0,$$

where ***J***_s_ is the surface flux. The surface divergence operator ∇_s_⋅ is defined as

2.2 $$\nabla_{s}\cdot a=\frac{\partial a^{i}}{\partial x^{i}}+\Gamma_{li}^{i}a^{l},$$

where ***x*** is the position vector in space and $\Gamma_{li}^{i}$ is the Christoffel symbol. The surface gradient operator ∇_s_=***P***∇, where ***P***=***I***−***n***⊗***n*** is the Laplace–Beltrami operator for an outward pointing unit normal to the interface ***n*** [44]. Using covariant curvilinear basis ***b***_*i*_ and contravariant curvilinear basis ***b***^*k*^:

2.3 $$\nabla_{s}a=\left[\frac{\partial a^{i}}{\partial x^{k}}+\Gamma_{lk}^{i}a^{l}\right]b_{i}⊗b^{k}.$$

Using Fick’s Law, the surface flux is related to the chemical potential *μ* via the surface gradient and a mobility *ν*( *f*) [43]:

2.4 $$J_{s}=-\nu (\;f)\nabla_{s}\mu .$$

From equations (2.1) and (2.4):

2.5 $$\frac{\partial f}{\partial t}-\nabla_{s}\cdot (\nu (\;f)\nabla_{s}\mu )=0.$$

Two types of mobility are considered in this work. The first type is constant mobility:

2.6 $$\nu (\;f)=\nu_{0},$$

where *ν*_0_ is the base surface mobility. This type of mobility is appropriate when molecules can freely move through bulk phases. The second type is more appropriate for situations where the majority of molecular motion occurs at the interface between phases. In this case, a degenerate mobility is defined as

2.7 $$\nu (\;f)=4\nu_{0}f(1-f).$$

It should be noted that, for the variable mobility case, the overall mobility in the bulk is very small, which is often the case in a realistic system [45,46]. It is also possible to use other mobilities, as has been recently investigated [47].

The chemical potential *μ* can be derived by applying the variational derivative to the free energy of the surface phase field [48]. Intuitively, it is expected that the local free energy will depend on the homogeneous free energy and the energy due to the interface separating the phases [10]. This energy functional can thus be written as

2.8 $$E[f]=\int_{\Gamma (x,t)}\left(g(\;f)+\frac{\epsilon^{2}}{2}{\left(\nabla_{s}\;f\right)}^{2}\right)dA.$$

The first term is the free energy of the homogeneous solution, while the second term is the interfacial energy, defined using the surface gradient of the concentration field, where *ϵ* is a constant associated with the domain interface energy. This form of energy functional is also known as the Landau–Ginzberg free energy functional [41]. Taking the variational derivative of the free energy functional with respect to a change in the concentration variable results in the chemical potential field [30,49]:

2.9 $$\mu =g^{\prime }(\;f)-\epsilon^{2}\Delta_{s}f,$$

where Δ_s_=∇_s_⋅∇_s_ is the surface Laplacian and *g*′( *f*) is the derivative of the mixing energy with respect to argument *f*.

The homogeneous free energy is usually described using a double-well potential. In this work, a simple mixing energy of

**Figure 1.**
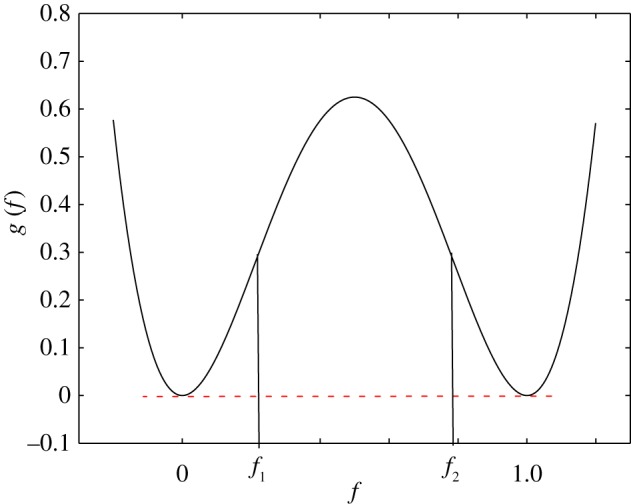
Homogeneous free energy of mixing.

2.10 $$g(\;f)=f^{2}{\left(1-f\right)}^{2},$$

as shown in figure 1, is used. The points *f*_1_ and *f*_2_ are called the spinodes, and are defined by ∂^2^*g*/∂^2^*f*=0. The region between by *f*_1_ and *f*_2_, given by ∂^2^*g*/∂*f*^2^<0, is known as the spinodal region, where a single phase decomposes into two phases. In this simple mixing energy, the equilibrium concentration of the two phases are defined by the two wells at concentrations *f*=0 and *f*=1.

To define the CHC equation, a white Gaussian noise [19,23] is added to the deterministic CH equation:

2.11 $$\frac{\partial f(x,t)}{\partial t}=\nabla_{s}\cdot (\nu \nabla_{s}\mu )+\xi (x,t),$$

where *ξ* denotes a stochastic Gaussian white noise dictated by the Fluctuation Dissipation Theorem with mean 〈*ξ*〉=0 and variance 〈*ξ*(***x***,*t*),*ξ*(***x***′,*t*′)〉=−2*νk*_B_*Tδ*(*t*−*t*′)Δ_s_*δ*(***x***−***x***′), where *δ* is the Dirac delta function [22,23]. The Fluctuation Dissipation Theorem implies that the random noise that is added is uncorrelated in time but partially correlated, specifically conserved, in space. The Laplacian in front of the Dirac delta function appears as this Langevin force term is present in a system that is characterized by conserved fields. These kinds of stochastic fluctuating partial differential equations are currently under investigation [50].

### 2.1. Non-dimensional system

The dimensionless CHC equation that governs the evolution of domains is

2.12 $$\frac{\partial f}{\partial \hat{t}}=\frac{1}{\mathrm{Pe}}{\hat{\nabla }}_{s}\cdot (\hat{\nu }{\hat{\nabla }}_{s}\hat{\mu })+\hat{\xi },$$

where the dimensionless units are represented by $\hat{\left(\cdot \right)}$ and Pe is the surface Peclet number, which relates the strength of any surface advection to diffusion. The dimensionless parameters are defined as follows:

2.13 $$\begin{array}{rl} & \hat{t}=\frac{t}{t_{0}},\;\hat{\nu }=\frac{\nu }{\nu_{0}},\;\hat{\mu }=\frac{\mu }{\mu_{0}},\\ & {\hat{\nabla }}_{s}=l_{0}\nabla_{s},\\ & \mathrm{Pe}=\frac{l_{0}^{2}}{t_{0}\mu_{0}\nu_{0}},\;{\mathrm{Cn}}^{2}=\frac{\epsilon^{2}}{\mu l_{0}^{2}}\\ \;\text{and}\; & \langle \hat{\xi }(x,t),\hat{\xi }(x^{\prime },t^{\prime })\rangle =-\sigma \hat{\nu }\delta (t-t^{\prime }){\hat{\Delta }}_{s}^{2}\delta (x-x^{\prime }), \end{array}\}$$

where *l*_0_ is the characteristic length, *t*_0_ is the characteristic time, *μ*_0_ is the characteristic chemical potential, *ν*_0_ is the characteristic mobility, Cn^2^ relates the ratio of the domain interface energy to the chemical potential, where Cn is called the Cahn number, and *σ* is the noise intensity defined as *σ*=(2*k*_B_*T*/*μ*_0_)^2^. Using this notation, the dimensionless mobility is now $\hat{\nu }=1$ for the constant mobility case and $\hat{\nu }=4f(1-f)$ for the variable mobility case. The dimensionless chemical potential equation is

2.14 $$\hat{\mu }=\frac{\partial \hat{g}}{\partial f}-{\mathrm{Cn}}^{2}{\hat{\Delta }}_{s}f.$$

Using equation (2.12) and equation (2.14), and dropping the $\hat{\left(\cdot \right)}$ notation the following fourth-order evolution equation for *f*(***x***,*t*) is obtained:

2.15 $$\frac{\partial f}{\partial t}+\frac{{\mathrm{Cn}}^{2}}{\mathrm{Pe}}\nabla_{s}\cdot (\nu \nabla_{s}\Delta_{s}f)=\frac{1}{\mathrm{Pe}}\nabla_{s}\cdot (\nu \nabla_{s}g^{\prime }(\;f))+\xi .$$

## 3. Numerical method

In this section, we discuss the numerical methods used to model phase dynamics on a curved surface. The interface is described using a level-set jet method [51,52]. To solve the surface evolution equation, we use the closest point method, which is described in §3b. We discretize the system using second-order, centred finite differencing techniques while a semi-implicit time discretization is employed.

### 3.1. Defining the curved surface using level sets

The level-set method is a tool to define and track an interface. Introduced by Osher & Sethian [53], this method has been used in a variety of applications including medical imaging [54], crystal growth [55], crack patterns [56] and semiconductor processing [57]. The idea is to define the interface implicitly through the use of an auxiliary mathematical function, akin to density, which allows for complex motion and topological changes such as merging and pinching. For details, readers are referred to Osher & Fedkiw [58] or Sethian & Smereka [59].

**Figure 2.**
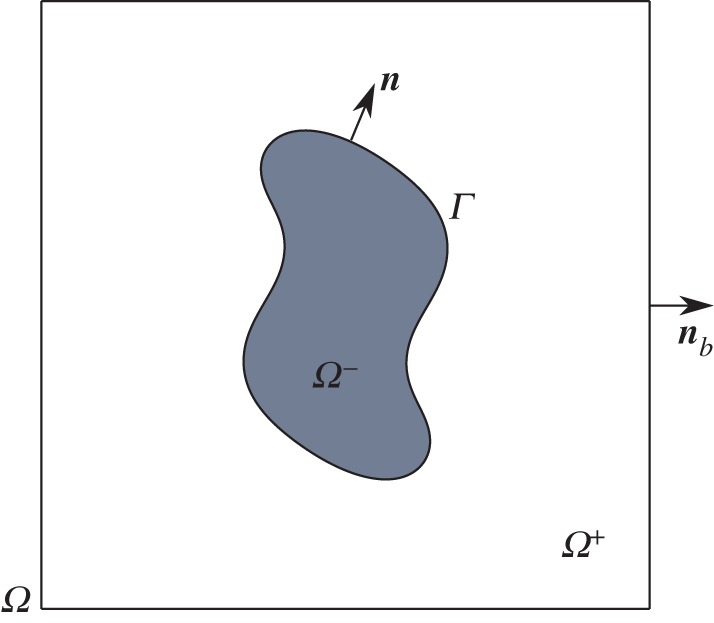
The computational domain.

Let *Γ*(***x***,*t*) be the interface separating regions *Ω*^−^ and *Ω*^+^. This interface is represented by the zero set of a higher-dimensional level-set function *ϕ*(***x***,*t*) (see figure 2):

3.1 Γ(x,t)={x:ϕ(x,t)=0}.

The region *Ω*^−^ is given by *ϕ*(***x***,*t*)<0, while *Ω*^+^ is defined as the region occupying *ϕ*(***x***,*t*)>0.

A significant advantage of this implicit representation is that many geometric quantities can easily be computed. For example, the normal and total curvature (sum of the principal curvatures) of the interface can be defined as

3.2 $$n=\frac{\nabla \phi }{∥\nabla \phi ∥}\;\mathrm{and}\;H=\nabla \cdot \frac{\nabla \phi }{∥\nabla \phi ∥}.$$

To model a surface differential equation accurate information must exist about the location of the interface. As the interface will, in general, not coincide with grid points, interpolation schemes must be used to determine the location of the interface. To aid in this, an extension of the base level-set method is used. The idea is to track not only a single level-set function *ϕ*, but also derivatives of the level set. A grouping of this information has been called a ‘jet’ of information [51]. Using this jet, it is possible to define high-order interpolants without the need for derivative approximations. For example, using a jet which consists of the level-set function *ϕ*, in addition to gradient vector fields, *ϕ*_*x*_ and *ϕ*_*y*_, and the first cross-derivative *ϕ*_*xy*_, it would be possible to define a cubic Hermite interpolant on a two-dimensional Cartesian grid without the need for derivative approximations. Additional information about the jet level-set method can be found in the work of Seibold *et al.* [51].

### 3.2. Phase field solver

The CHC system, equation (2.15), can be written as a pair of coupled, second-order differential equations [42,43]:

3.3 $$\frac{\partial f}{\partial t}-\frac{1}{\mathrm{Pe}}\nabla_{s}\cdot (\nu \nabla_{s}\mu )-\xi =0\;\text{and}\;\mu +{\mathrm{Cn}}^{2}\Delta_{s}f=g^{\prime }(\;f).$$

Here, a second-order backward-finite-difference (BDF2) scheme [60] is used to discretize in time. The system can then be written as

3.4 $$\left[\begin{array}{cc} I & {\mathrm{Cn}}^{2}L_{s}\\ -\frac{2\Delta t}{3\mathrm{Pe}}L_{s}^{\nu } & I \end{array}\right]\left[\begin{array}{c} \mu^{n+1}\\ f^{n+1} \end{array}\right]=\left[\begin{array}{c} g^{\prime }(\hat{f})\\ \frac{4}{3}f^{n}-\frac{1}{3}f^{n-1}+\frac{2}{3}\Delta t\xi^{n} \end{array}\right],$$

where *Δt* is a fixed time step. In the above block system, ***I*** is the identity matrix, the constant-coefficient surface Laplacian is given by ***L***_s_≈Δ_s_ and the variable-coefficient surface Laplacian is given by ***L***^*ν*^_s_≈∇_s_⋅(*ν*∇_s_). The solutions ***f***^*n*^ and ***f***^*n*−1^ are at times *t*^*n*^ and *t*^*n*−1^, respectively, and the approximation to the solution at time *t*^*n*+1^ is given by $\hat{f}=2f^{n}-f^{n-1}$.

As this is a surface partial differential equation, specialized methods are required to evolve the system properly. In this work, the closest point method is used. The closest point method was first developed and analysed by Ruuth & Merriman [61] and has been modified to increase numerical stability and accuracy [62]. The basic idea is to extend the solution to a surface differential equation away from the interface such that it is constant in the normal direction. With this extension, it is possible to write a surface differential equation as a standard differential equation in the embedding space. It has been previously shown that the surface Laplacian operator can be computed with second-order accuracy using linear and cubic polynomial interpolations [63].

Let ***E***_1_ be a linear polynomial interpolation operator and ***E***_3_ be a cubic polynomial interpolation operator. For any point ***x*** not on the interface these operators return the value of a function at the interface point closest to ***x***. For example, the operation ***E***_3_***f*** returns the value of *f* at the point on the interface closest to ***x*** using the cubic interpolation function. Using this notation, the block matrix in equation (3.4) is rewritten as

3.5 $$\begin{array}{rl} & \left[\begin{array}{cc} I & {\mathrm{Cn}}^{2}[E_{1}L+\alpha (E_{3}-I)]\\ -\frac{2\Delta t}{3\mathrm{Pe}}[E_{1}L^{\nu }+\alpha (E_{3}-I)] & I \end{array}\right]\left[\begin{array}{c} \mu^{n+1}\\ f^{n+1} \end{array}\right]\\ & \;=\left[\begin{array}{c} g^{\prime }(\;\hat{f})\\ \frac{4}{3}\;f^{n}-\frac{1}{3}f^{n-1}+\frac{2}{3}\Delta t{\tilde{\xi }}^{n} \end{array}\right], \end{array}$$

with *α*=6/*h*^2^, where *h* is the uniform grid spacing, and ***L***≈Δ represents the Cartesian finite difference approximation to the constant standard Laplacian and $L^{\nu }\approx \nabla \cdot (\tilde{\nu }\nabla )$ represents the Cartesian finite difference approximation to the variable-coefficient Laplacian. Quantities denoted with $\left(\tilde{\cdot }\right)$ indicate that the value has been extended off the interface. The addition of the *α* term, also known as a side condition, ensures that the solutions are constant in the normal direction. If this extension holds, then surface operators can be replaced with standard Cartesian operators. See Chen & Macdonald for complete details [63].

The block system shown in equation (3.5) is solved using the preconditioned Flexible GMRES algorithm available in PETSc [64–66]. The preconditioner is based on an incomplete Schur complement. Let ***L***_*E*_=***E***_1_***L***+*α*(***E***_3_−***I***) and $L_{E}^{\nu }=E_{1}L^{\nu }+\alpha (E_{3}-I)$. The preconditioner is then

3.6 $$P=\left[\begin{array}{cc} I & -{\mathrm{Cn}}^{2}L_{E}\\ 0 & I \end{array}\right]\;\left[\begin{array}{cc} I & 0\\ 0 & {\hat{S}}^{-1} \end{array}\right]\;\left[\begin{array}{cc} I & 0\\ \frac{2\Delta t}{3\mathrm{Pe}}L_{E}^{\nu } & I \end{array}\right].$$

The Schur complement is written as $S=I+(2{\mathrm{Cn}}^{2}\Delta t/3\mathrm{Pe})L_{E}L_{E}^{\nu }$. The application of the approximate Schur complement inverse, ${\hat{S}}^{-1}$, is obtained via five iterations of an algebraic multigrid preconditioning method [67].

### 3.3. Noise calculation

The noise term is calculated based on the Fluctuation Dissipation Theorem as follows:

3.7 $$\xi (x,t)=N(0,-\sigma \nu \delta (t-t^{\prime })\Delta_{s}\delta (x-x^{\prime })).$$

Writing the mean and the variance in discrete form:

3.8 $$\langle \xi_{x_{\Gamma }}^{n}\rangle =0$$

and

3.9 $$\langle \xi_{x_{\Gamma }}^{n}\xi_{y_{\Gamma }}^{n+1}\rangle =-\sigma \nu (\;f_{x_{\Gamma }}^{n})\frac{\delta_{\left(n)(n+1\right)}}{|t^{n}-t^{n+1}|}\Delta_{s}^{h}\frac{\delta_{\left(x_{\Gamma })(y_{\Gamma }\right)}}{h^{2}},$$

where ***x***_*Γ*_ and ***y***_*Γ*_ are two different points on the interface and *n* defines the time step [22]. As we are considering a two-dimensional surface, the grid size *h* is raised to the second power [25]. The proof of the discretization in the above can be found in [68].

To compute the random forcing term, $\tilde{\xi }$, the following procedure is used. To ensure consistency of the scheme this forcing term must be constant in the normal direction. This can be accomplished by computing the random force contribution at the closest point of any grid point and extending this quantity outwards. At a closest point, a random tangential vector is determined by choosing two random numbers, *ρ*_1_ and *ρ*_2_, from a Gaussian distribution with zero mean and unit variance. A random surface vector is then determined by ***ρ***=*ρ*_1_***t***+*ρ*_2_***b***, where ***t*** and ***b*** are two orthonormal vectors on the surface, such as the principal directions. Once these tangential random vectors are calculated in a region around the interface, it is possible to define the random force through

3.10 $$\tilde{\xi }=\sqrt{\frac{\sigma \nu (\;f)}{h^{2}\Delta t}}\nabla_{s}\cdot \tilde{\rho },$$

where a constant time step, *Δt*, is assumed. Note that as the surface Laplacian is approximated numerically, it may fail to preserve the fluctuation dissipation balance in the exact sense.

### 3.4. Conservation of surface phase concentration

After solving the system of the partial differential equation, equation (3.4), there will be certain amount of loss of surface phase concentration owing to numerical diffusion. The accumulative effect may have a drastic change on the average surface concentration over time. There have been numerous attempts to fix this issue in the past; see [69–71], for examples. In this work, a correction method is implemented. This method was introduced by Xu *et al.* [72], with the idea of adjusting the surface phase concentration at the end of every time step to ensure mass conservation. Let *f*_*h*_, *ϕ* and *Γ* be the solution of the discrete surface phase concentration equation (3.5), level set and interface at a given point in time, and let *f*_0_, *ϕ*_0_ and *Γ*_0_ be the initial phase concentration, initial level-set function and initial interface, respectively. Then, a surface phase concentration conservation parameter, *β*, is chosen such that the following condition is true:

3.11 $$\int_{\Gamma }\beta f_{h}\;dA=\int_{\Gamma_{0}}f_{0}\;dA.$$

Hence, *β* is computed as

3.12 $$\beta =\frac{\int_{\Gamma_{0}}f_{0}\;dA}{\int_{\Gamma }f_{h}\;dA}=\frac{\int_{\Omega }f_{0}\delta (\phi_{0})\;dV}{\int_{\Omega }f_{h}\delta (\phi )\;dV},$$

where *δ* is the Dirac delta function and the integrals are now performed over the embedding domain. The surface phase concentration is then modified at each time step as *f*=*βf*_*h*_. For further details, we refer the reader to Xu *et al.* [72].

## 4. Results on a sphere

In this section, qualitative and quantitative results are presented using the method described in previous sections. First, the sample evolution of phase dynamics is examined. Following that, a quantitative analysis on the domain dynamics is performed. This includes a convergence study to justify the grid size and time step used for the analysis. The growth rate of the domains is examined, and in particular the impact of variable and constant mobility in the system is considered. Finally, the role of noise in the system is investigated.

**Figure 3.**
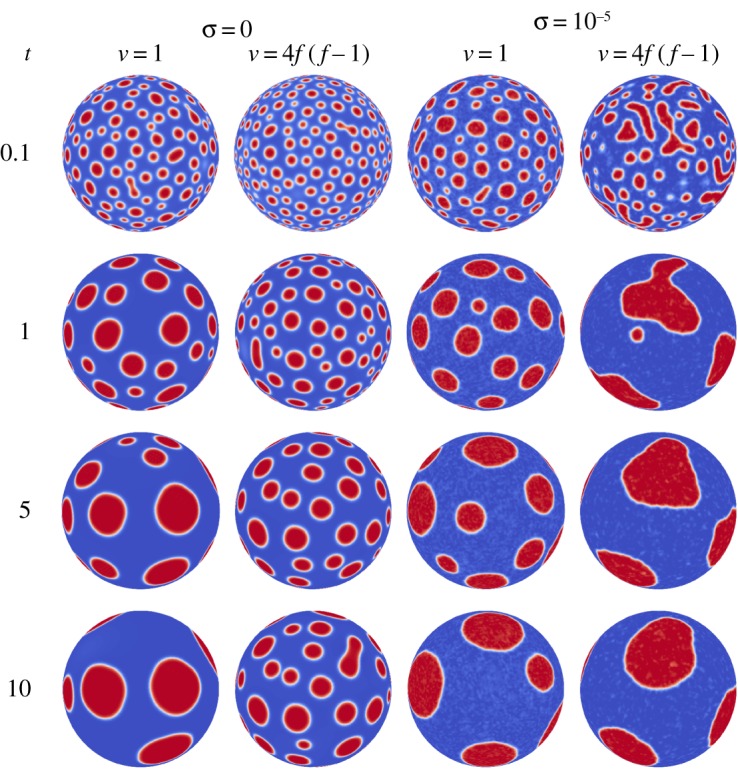
Evolution for the CH (*σ*=0) and CHC (*σ*=10^−5^) models with constant and variable mobility.

For simplicity, the shape considered is a unit sphere in a computational domain spanning [−1.25,1.25]^3^. Unless otherwise stated, the average concentration is set to 0.3, with an initial random perturbation of 0.01. The Peclet number is set to Pe=1.0, the Cahn number is Cn=0.015, and when noise is present it has an intensity of *σ*=10^−5^. See figure 3 for a sample evolution.

Surface dynamics will be quantified by a characteristic length, $\bar{R}(t)$, for the domains present on the surface. This surface characteristic length is defined as

4.1 $$\bar{R}(t)=\frac{A(t)}{L(t)},$$

where $A(t)=\int_{\Gamma }f\;dA$ is the total area of the domains and $L(t)=\int_{\Gamma }∥\nabla_{s}f∥\;dA$ is the corresponding total interface length of the domains.

### 4.1. Sample evolution of phase dynamics

In this section, the dynamics on a smooth spherical surface is examined for four cases: (i) CH with constant mobility, (ii) CH with variable mobility, (iii) CHC with constant mobility, and (iv) CHC with variable mobility. In all four cases, the initial condition is a random perturbation with a magnitude of 0.01 about the average concentration of 0.3. There are three expected regimes. Initially, very rapid phase segregation will occur and a large number of domains will appear. This will be followed by slow coarsening of the domains, which results in an increasing average domain size. The final regime will be characterized by a very slow coarsening process. In the simulations performed in this work, approximately 400 domains are seen during the initial phase segregation process. These domains coarsen in time, and only five to six domains remain in the final slow coarsening stage. See figure 3 for a sample evolution.

For the case of the CH model with constant mobility, the fast phase segregation occurs up to a time of *t*=0.1 (see figure 3), thereafter the domains start to slowly coarsen in time. The primary means of coarsening in this case is Ostwald ripening, where a domain large in size grows at the expense of smaller, nearby, domains. In this type of behaviour, the centre of each domain remains relatively fixed. The use of degenerate mobility decreases this coarsening rate but does not change the coarsening mechanism, which can be seen by comparing the first two columns of figure 3.

The combination of CHC and variable mobility has the effect of increasing the coarsening rate. This can be observed by visually comparing the sizes of the domains at a time of *t*=1, as the general size of the domains in the CHC plus variable mobility are larger than the other three cases. This behaviour will be further explored in subsequent sections.

### 4.2. Convergence study

**Figure 4.**
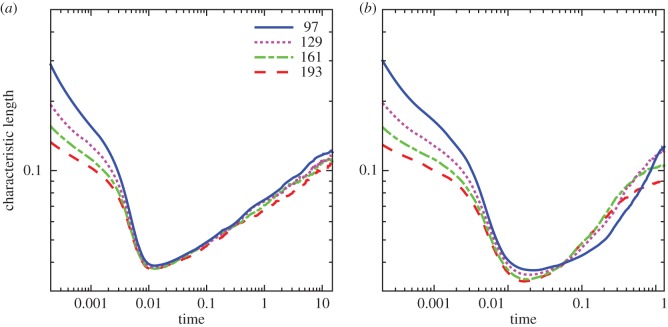
The characteristic length over time using the CHC model on a sphere with constant and variable mobilities for various grid sizes. (*a*) Constant mobility and (*b*) variable mobility.

The numerical convergence of the CH system in the absence of noise has been previously investigated by Gera & Salac [73]. In this section, a qualitative convergence study is performed with regard to the change of the characteristic length over time. Sample plots of the characteristic length over time for both the constant and variable mobility case are shown in figure 4. Here, four different grid sizes are considered: *N*=97, *N*=129, *N*=161 and *N*=193. For each case, the time step is set to *Δt*=5.12×10^−3^*h*, where *h*=2.5/(*N*−1) is the grid spacing.

Initially there is a rapid decrease in the characteristic length as the system undergoes rapid phase segregation from a well-mixed interface to one with many, small domains. After this point, the domains coarsen at a given rate, before reaching the near-equilibrium configuration. This middle region, after initial coarsening and before the near-equilibrium dynamics, is the region of interest.

For the constant mobility case (figure 4*a*), the growth is in this middle region, from approximately *t*=0.1 to *t*=10, and the rate is similar across all grid spacings. With variable mobility, figure 4*b*, the region of interest is only from *t*=0.1 to *t*=1, as the final very slow coarsening stage is achieved sooner. A larger difference between the *N*=97 grid compared to the others is seen. There is little qualitative difference in the growth rates using grid sizes larger than *N*=129, and thus that is the size chosen for further analysis.

### 4.3. Characteristic length and energy evolution

In this section, sample evolution curves for the characteristic length (equation (4.1)) and the total energy of the system (equation (2.8)) are examined over time for the CH and CHC systems, assuming both constant and variable mobility. To explore the CHC systems, three simulation results for each mobility case is shown. These will then be compared to a single CH simulation. As was mentioned earlier, the noise intensity level for the results in this section is *σ*=10^−5^.

**Figure 5.**
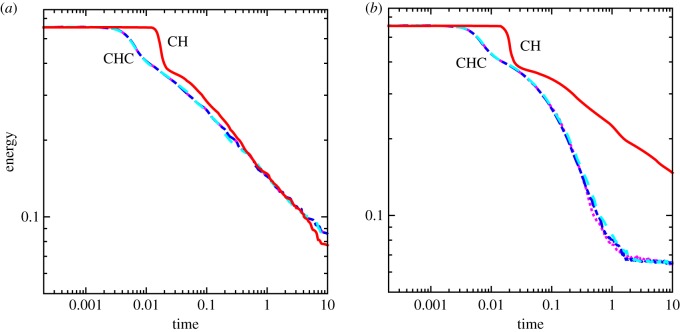
The change of total energy in the CH model along with the three sample runs of a CHC model for constant and variable mobilities. (*a*) Constant mobility and (*b*) variable mobility.

**Figure 6.**
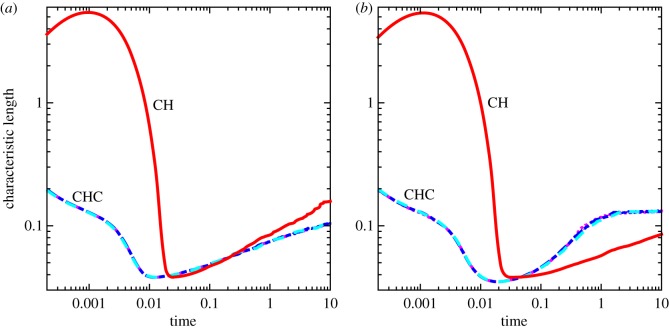
The characteristic length over time for the CH model along with three sample runs of the CHC model for constant and variable mobilities. (*a*) Constant mobility and (*b*) variable mobility.

The energy (figure 5) and characteristic length (figure 6) are shown over time for the single CH simulation and three representative CHC simulations. During the initial stage when the system is in a homogeneous state, the total energy is large and remains constant for both the CH and CHC systems. After some time the initial state segregates into many, small domains; see figure 3 for an example. During this rapid phase segregation regime, both the CH and CHC simulations see an overall decrease in the characteristic length, eventually reaching a minimum length, while a rapid decrease in the overall energy occurs. In both mobility cases, the CHC model begins the segregation process earlier, as is evident from the earlier decrease of the energy. It is also interesting to note that the CH system has a brief increase in the characteristic length for both constant and variable mobility. This can be attributed to the CH system remaining near the well-mixed initial condition longer than the CHC system. Instead of quickly segregating to well-defined domains, the CH simulations have many, small amplitude fluctuations. This results in a relatively small value of *L*(*t*), which quickly increases as the domains form.

After this rapid phase segregation, the system undergoes a steady and much slower coarsening process. For the constant mobility case (figures 5*a* and 6*a*), it is clear that the growth rate for the CHC system is below that of the CH system. At a time of *t*=10, the energy of the system is higher and the characteristic length is smaller for the CHC system when compared with the CH system. Thus, while noise promotes the early start of the coarsening process when constant mobility is assumed, it inhibits the process during the slower, second coarsening regime. It should also be noted that as the size of the domains becomes large compared to the radius of the underlying shape, the coarsening rate may further decrease.

The assumption of variable mobility dramatically changes the influence of the noise (see figures 5*b* and 6*b*). While the CHC system begins to segregate earlier than the CH system, similar to the constant mobility case, the rate of change of CHC with variable mobility is much higher than CH with variable mobility. It is suspected that the different scalings of the noise magnitude with respect to the mobility is the cause of this behaviour. This will be further explored in §4.4.

### 4.4. Discussion and analysis

**Figure 7.**
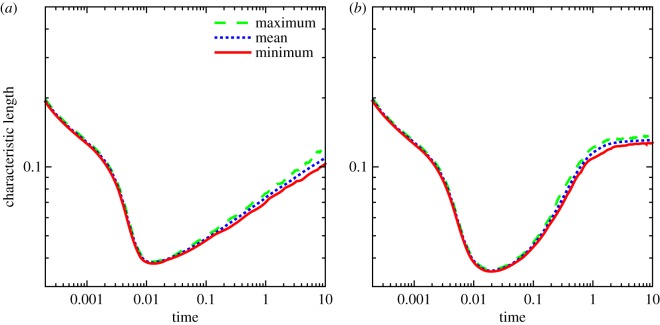
The minimum, maximum and mean characteristic lengths for the CHC system for the 64 realizations using constant and variable mobilities each. (*a*) Constant mobility and (*b*) variable mobility.

To further explore the CH and CHC systems, a total of 64 realizations (simulations) per noise level and mobility type have been performed. For the CHC system with *σ*=10^−5^, the minimum, maximum and average characteristic lengths for each time step have been determined from the 64 realizations, as shown in figure 7. Owing to the cumulative effects of the noise during the course of the simulation, the spread of the characteristic length increases as time progresses.

After the initial segregation phase, it is expected that the characteristic length grows at a particular growth rate, $\bar{R}(t)\propto t^{\alpha }$, where *α* is the growth rate. For CH and CHC with constant mobility, in addition to the variable mobility CH model, this region extends from approximately a time of *t*=0.1 to *t*=10. Owing to the faster dynamics of the variable mobility CHC model, this region exists approximately from *t*=0.1 to *t*=0.8. After these times the system enters the long-term, slow growth phase. To determine the growth rate, a linear fit is made to the appropriate region. The slope of this fit is taken to be the growth rate parameter *α*. The complete results for the mean, standard deviation and coefficient of variation for all considered systems are presented in table 1.

**Table 1.** Statistics on the growth rate for the CH and CHC models with constant and variable mobilities.

model	mobility	noise	mean	s.d.	coefficient of variation
CH	1	—	0.2814	0.0215	0.0764
	*f*(1−*f*)	—	0.1759	0.0062	0.0352
CHC	1	10^−9^	0.2825	0.0197	0.0697
		10^−7^	0.2672	0.0241	0.0902
		10^−5^	0.1760	0.0134	0.0761
	*f*(1−*f*)	10^−9^	0.1765	0.0092	0.0521
		10^−7^	0.3719	0.0148	0.0398
		10^−5^	0.4278	0.0154	0.0360

For the CH system, the growth rate for constant mobility was determined to be $\bar{\alpha }=0.2814$, which differs from the theoretical growth rate of $\alpha =\frac{1}{3}$ for flat surfaces [74,75]. This deviation from the theory indicates that the underlying geometry does have an impact on the rate at which phase segregation occurs. In this case, the curvature of the sphere has played a role in retarding the rate of coarsening. When a degenerate mobility is used, this growth rate decreases to a value of $\bar{\alpha }=0.1759$. As mentioned earlier, this decrease should be expected as the evolution process is now limited to only occur near the interface.

The CHC system is explored by not only varying the mobility type, but also the intensity of the noise, *σ*. First, consider the constant mobility case. Using a noise intensity of *σ*=10^−5^, the average growth rate decreased to a value of $\bar{\alpha }=0.1760$. As the noise intensity decreases, the mean approaches that of the CH system, with values of $\bar{\alpha }=0.2672$ for *σ*=10^−7^ and $\bar{\alpha }=0.2825$ for *σ*=10^−9^. This trend of recovering the CH system as the noise intensity is lowered has been also observed in the past [19].

When variable mobility is employed, the mean growth rate of a CHC model increases to $\bar{\alpha }=0.4278$ when *σ*=10^−5^. As the noise intensity level decreases, the growth rate also decreases, with $\bar{\alpha }=0.3719$ for *σ*=10^−7^ and $\bar{\alpha }=0.1765$ for *σ*=10^−9^. As with the constant mobility case, this growth rate approaches the CH value.

The fact that the growth rate increases for CHC and variable mobility is quite surprising. One possible explanation can be obtained by comparing two contributions to ∂*f*/∂*t*: the diffusive contribution $\nu \Delta_{s}^{2}f$ and the conserved random force *ξ*. The diffusive contribution will tend to smooth out any oscillations which occur, while the random force will push the system away from an equilibrium configuration.

Consider a simple, one-dimensional equilibrium phase field profile, which is given by Lee *et al*. [76]:

4.2 $$f_{\mathrm{eq}}(x)=\frac{1}{2}\left[\tanh\left(\frac{x}{\mathrm{Cn}\sqrt{2}}\right)+1\right].$$

**Figure 8.**
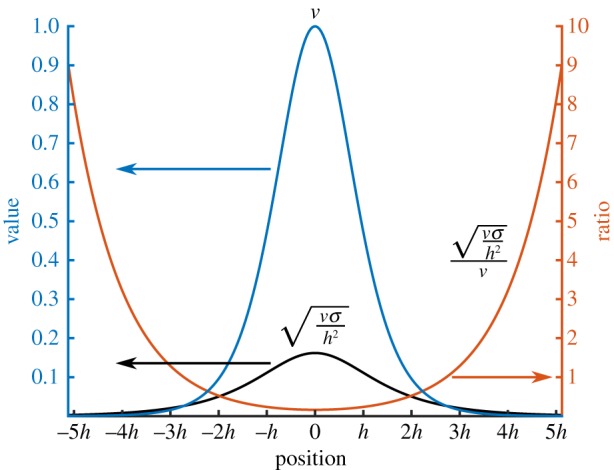
The mobility (*ν*=4*f*(1−*f*)) and noise magnitude ($\sqrt{\nu \sigma /h^{2}}$), where *σ*=10^−5^ and *h*=2.5/128 for an equilibrium one-dimensional profile, equation (4.2). The ratio of the noise magnitude to mobility is also provided.

The diffusive contribution scales as the mobility, *ν*, while at the discrete level the noise will scale as $\sqrt{\nu \sigma /h^{2}}$ (equation (3.10)). As an example use Cn=0.015 with *σ*=10^−5^ and $h=\frac{2.5}{128}$. The value of the mobility and of the noise scaling is presented in figure 8. The ratio between the noise scaling and the mobility is also shown in this figure.

Both the mobility and noise magnitude decrease quickly away from *x*=0, with the mobility decreasing at a faster rate than the noise magnitude. This becomes apparent when the ratio is considered. In regions away from *x*=0, the influence of noise becomes larger than the diffusive term. It is suspected that the larger influence of noise in regions away from the interface drives the system to coarsen faster than the other cases. This behaviour also explains the oscillations (faint white patches) observed in the well-segregated regions in figure 3, as the influence of the noise is relatively large, compared to the diffusive terms. Note that other forms of mobility, for example, one which does not decrease as quickly away from the interface, may result in different dynamics.

**Figure 9.**
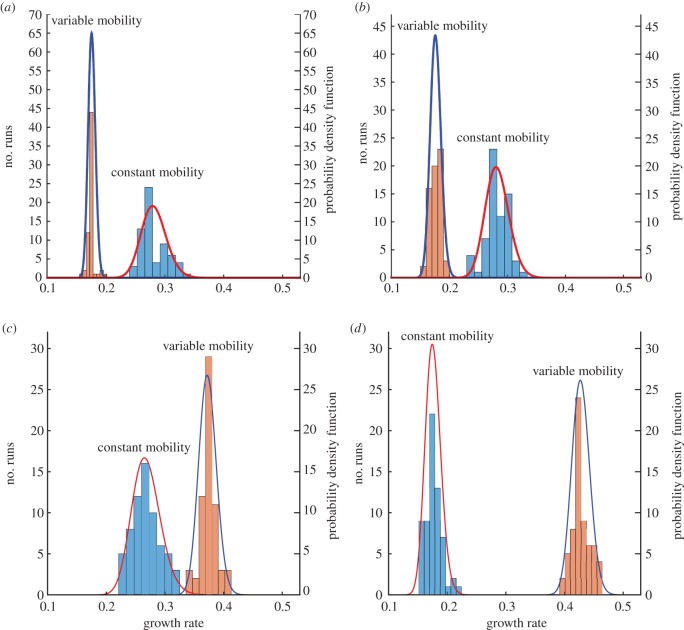
Histograms for the growth rate of the characteristic length for CH and CHC systems using 64 realizations for constant and variable mobilities. A lognormal distribution function is fitted on the results. (*a*) CH, (*b*) CHC: *σ*=10^−9^, (*c*) CHC: *σ*=10^−7^ and (*d*) CHC: *σ*=10^−5^.

The complete CH and CHC results for various noise intensity levels can be seen in the histograms shown in figure 9. Using these data, a probability density function is fitted and also shown on the histograms. As the growth rate can never be negative, only non-negative distributions were explored. The lognormal distribution gave a good qualitative fit and as the null hypothesis also passed the Kolmogorov–Smirnov test at 5% level of significance [77] for all situations, it is chosen to be an appropriate fit.

The probability density function of the lognormal distribution is given by

4.3 $$f(x\;|\;\mu ,\sigma_{\mathrm{pdf}})=\frac{1}{x\sigma_{\mathrm{pdf}}\sqrt{2\pi }}\exp\left(\frac{-{\left(\ln x-\mu \right)}^{2}}{2\sigma_{\mathrm{pdf}}^{2}}\right)\;\text{with}\;x>0,$$

where *x* is the data, *μ* is the log mean, and *σ*_pdf_ is log standard deviation with the support −∞<μ<∞ and *σ*_pdf_≥0. The log mean and the log standard deviation can be estimated from the probability density function. See table 2 for the fitted parameters. It is noted as the noise intensity goes down the log mean value approaches the result for the CH system.

**Table 2.** Parameter estimates for the lognormal distribution function that fits the histogram of the growth rate for the CH and CHC systems with constant and variable mobilities.

model	mobility	noise	*μ*	*σ*_pdf_
CH	1	—	−1.2707	0.0748
	*f*(1−*f*)	—	−1.7384	0.0349
CHC	1	10^−9^	−1.2664	0.0716
		10^−7^	−1.3237	0.0902
		10^−5^	−1.7339	0.0747
	*f*(1−*f*)	10^−9^	−1.7358	0.0523
		10^−7^	−0.9900	0.0402
		10^−5^	−0.8498	0.0357

## 5. Dumbbell interface

In this section, the phase segregation on a dumbbell is examined. The shape are two spheres connected by a cylinder. Each sphere has a radius of 0.75 and are centred at (−1.125,0,0) and (1.125,0,0), while the cylinder connecting the two spheres has a radius of 0.375. The average concentration is 0.3, while the initial random perturbation has a magnitude of 0.01. To focus on the influence of the underlying geometry, only the constant-mobility case is considered.

**Figure 10.**
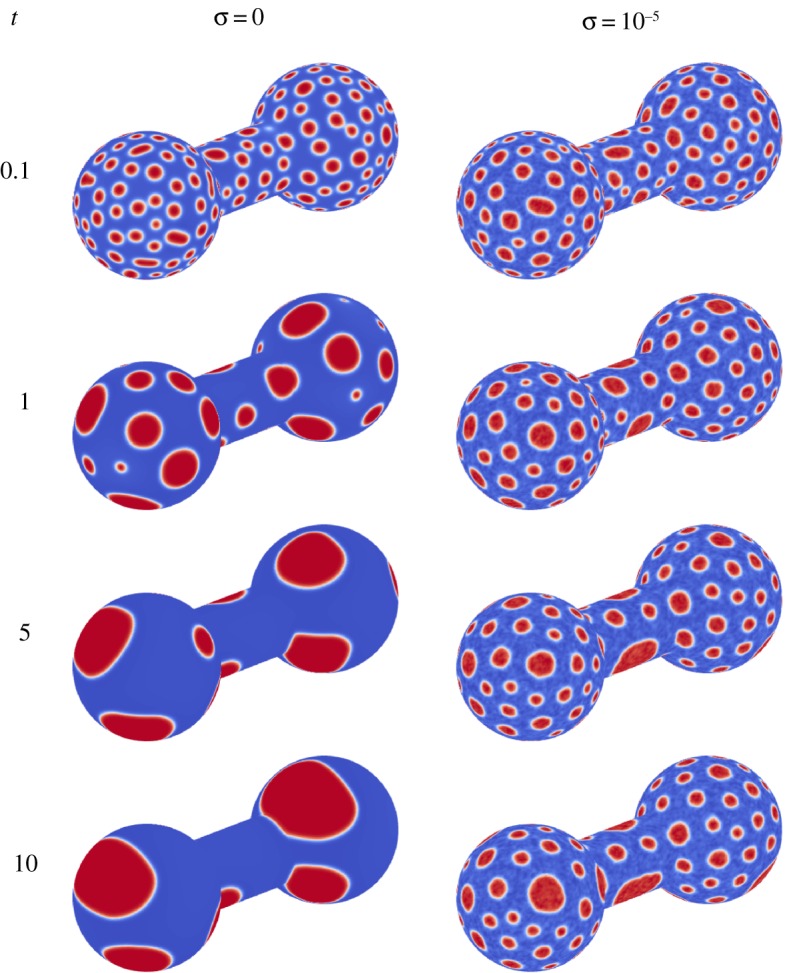
Evolution for CH (*σ*=0) and CHC (*σ*=10^−5^) models with constant mobility on a dumbbell.

First, consider the CH system (figure 10). As before, the initially well-mixed system quickly segregates into many, small domains. Over time, the domains begin to grow and coarsen, until a small number of large domains exist. Next, consider the CHC system with *σ*=10^−5^ (again figure 10). As with the CH system, the well-mixed system quickly segregates into small domains. Unlike the CH case, the growth rate of the domains using the CHC model on the dumbbell is greatly reduced, as seen by the many small domains at a time of *t*=10. This indicates that the underlying geometry has a large influence on the coarsening process in the presence of noise.

**Figure 11.**
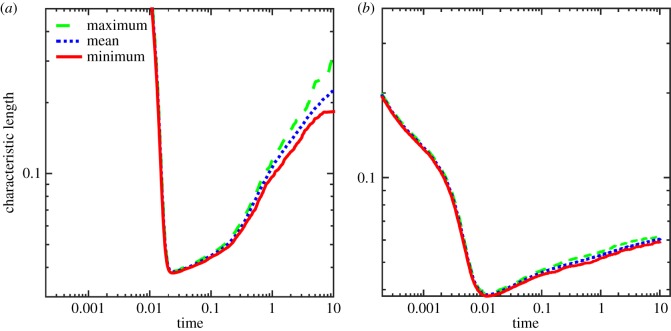
The minimum, maximum and mean characteristic lengths for the CH and CHC systems for the 64 realizations using constant mobility on a dumbbell. (*a*) CH model and (*b*) CHC model.

**Figure 12.**
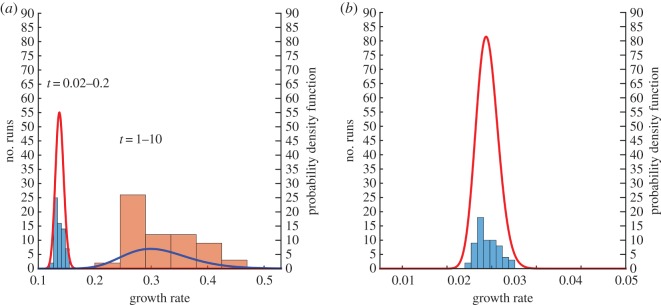
Histograms for the growth rate of the characteristic length for CH and CHC systems using 64 realizations for constant mobility on a dumbbell. A lognormal distribution function is fitted on the results. (*a*) CH and (*b*) CHC: *σ*=10^−5^.

As before, a statistical analysis of the growth rate is carried out for the CH and the CHC models with *σ*=10^−5^, using 64 realizations for each model. The minimum, maximum and average characteristic length for each time step is plotted in figure 11. As expected, the characteristic length for both the CH and CHC model increases over time, with the spread between the minimum growth rate and the maximum growth rate also increasing with time. Additionally, the mean, standard deviation and coefficient of variation are presented in table 3, while the corresponding histograms are shown in figure 12.

**Table 3.** Statistics on the growth rate for the CH and CHC models with constant mobility of a dumbbell.

model	time	noise	mean	s.d.	coefficient of variation
CH	0.02–0.2	—	0.1380	0.0073	0.0529
	1–10	—	0.3152	0.0607	0.1926
CHC	0.1–10	10^−5^	0.0581	0.0050	0.0861

**Figure 13.**
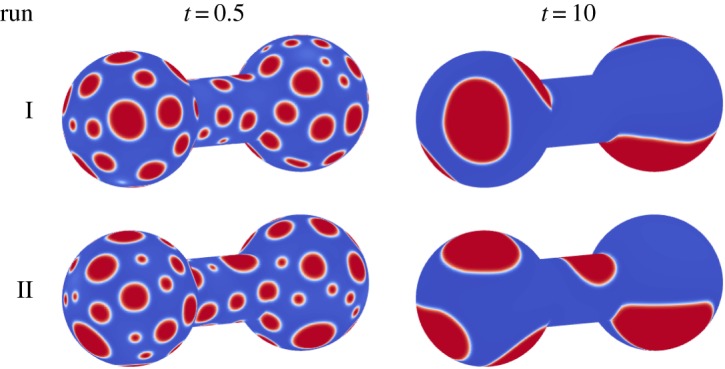
Evolution for the CH model with constant mobility on a dumbbell, for two realizations.

When considering the CH model, there are two growth rates apparent in the system. The first extends from *t*=0.02 to *t*=0.2, while the second extends from *t*=1.0 onwards. It is, therefore, appropriate to consider two growth rates, with $\bar{\alpha }=0.1380$ from *t*=0.02 to *t*=0.2 and $\bar{\alpha }=0.3152$ from *t*=1.0 onwards. When considering this latter regime, the standard deviation and coefficient of variation are quite large, as presented in table 3. It is suspected that the underlying interface plays a large role in the variation of the growth rate at late times. To demonstrate this, consider two sample runs shown in figure 13. At a time of *t*=0.5, both interfaces are well covered by small domains, with the distance between domains similar. At a time of *t*=10, the domains for run I are predominantly on the two spheres, with no domain on the connecting cylinder. In run II, there is a domain on the cylinder. The distance between domains plays a critical role in the coarsening process, with a larger distance corresponding to a lower growth rate. Unlike the spherical interface, the lack of full symmetry in the dumbbell shape results in a growth rate which depends on the initial condition, as that will determine where large domains will preferentially occur.

Now, consider the CHC system with a noise intensity of 10^−5^. From the sample result in figure 10 and table 3, it is apparent that the growth rate is very small, with a value of $\bar{\alpha }=0.0581$ and a small standard deviation of 0.005. It is suspected that the larger curvatures present in the dumbbell shape, coupled with the large noise intensity, result in this decrease in the growth rate.

## 6. Conclusion

In this work, the CHC model is solved on smooth interfaces using a splitting method that converts the fourth-order partial differential equation into two coupled second-order partial differential equations. The surface differential equations are solved using the closest point method, using a level-set jet scheme to describe the interface.

These results indicate that the underlying surface plays a large role in the segregation process, both in the presence and in the absence of thermal fluctuations/noise. When assuming constant surface mobility, the presence of noise slows the coarsening rate of domains, with the growth rate increasing with a decrease in the noise magnitude. Surprisingly, the presence of noise actually increases the growth rate when assuming a degenerate mobility. This is most probably owing to the fact that the diffusive evolution contributions decay at a faster rate than the noise contributions as one moves away from the interface.

When examining a spherical interface in the absence of noise and constant mobility, the overall growth rate is slightly lower than that predicted for flat, two-dimensional surfaces. The inclusion of noise further decreases the observed growth rate. The use of a dumbbell shape, with a spatially varying curvature, further influences the evolution. Assuming no noise, two growth regimes were identified on the dumbbell, with the final growth rate highly dependent on the initial condition. Inclusion of noise for the dumbbell shape dramatically decreased the growth rate.

In this work, it has been established that the underlying curvature of the surface does play a role in domain coarsening. For a CH case with constant unit mobility, the growth rate remains close to $\frac{1}{3}$. Surfaces with negative Gaussian curvatures, or surfaces with higher genus are expected to coarsen in a similar manner. However, when noise is added and mobility is no longer constant, varying behaviours can be expected. The authors will formally investigate this in future work.

## Data accessibility

The simulation code is stored at https://bitbucket.org/dsalac/3d-vesicle.
